# Cholinesterase inhibitors for the treatment of dementia: real-life data in Hungary

**DOI:** 10.1007/s11357-021-00470-7

**Published:** 2021-10-15

**Authors:** Nóra Balázs, Dániel Bereczki, András Ajtay, Ferenc Oberfrank, Tibor Kovács

**Affiliations:** 1 Department of Neurology, Semmelweis University, Balassa utca 6., H-1083 Budapest, Hungary; 2 MTA-SE, Neuroepidemiological Research Group ELKH, Balassa utca 6., H-1083 Budapest, Hungary; 3 Institute of Experimental Medicine, Szigony utca 43., H-1083 Budapest, Hungary

**Keywords:** Alzheimer's disease, Dementia, Cholinesterase inhibitors, Epidemiology, Pharmacoepidemiology

## Abstract

Dementia is one of the leading causes of death and disability in older population. Previous reports have shown that antidementia medications are associated with longer survival; nonetheless, the prevalence of their use and the compliance with them are quite different worldwide. There is hardly any available information about the pharmacoepidemiology of these drugs in the Eastern-European region; we aimed to analyze the use of cholinesterase inhibitors (ChEis) for the treatment of dementia to provide real-life information from the Eastern European region. All medical and medication prescription reports of the in- and outpatient specialist services collected in the NEUROHUN database in Hungary were analyzed between 2013 and 2016. Survival, adherence, and persistence values were calculated. 8803 patients were treated with ChEis during the study period, which was only 14.5% of the diagnosed demented patients. The survival of treated patients (more than 4 years) was significantly longer than patients without ChEi treatment (2.50 years). The best compliance was observed with rivastigmine patch. Choosing the appropriate medication as soon as possible after the dementia diagnosis may lead to increased life expectancy.

## Introduction

Dementia is a clinical syndrome characterized by progressive cognitive decline interfering with the ability to function independently [1]. The morbidity and mortality of dementia is increasing worldwide, being one of the leading causes of death and disability in older population [2]. Alzheimer’s disease (AD) is the most common cause of dementia, accounting for 60–80% of all cases [3]. Currently, only symptomatic treatment is possible; disease-modifying pharmacotherapies have not been developed yet. Cholinesterase inhibitors (ChEis) (such as donepezil, rivastigmine, and galantamine) and the *N*-methyl-d-aspartate receptor antagonist memantine are the available antidementia drugs for AD. Use of ChEis results temporary improvement in cognitive functions, in global clinical state, in activities of daily living, and in behavioral and psychological symptoms of dementia (BPSD) [3]. In addition, the use of rivastigmine is also approved in mild to moderate Parkinson's disease dementia [3], but ChEis could be beneficial in other types of dementias such as vascular (VaD) or Lewy body dementia as well [4, 5].

More than half of the people over the age of 65 should take five or more medications regularly for chronic diseases and more than half of these patients had complication to follow the recommendation of their physicians, leading to serious healthcare and economic consequences [6]. Symptoms of dementia make it even more difficult to take antidementia drugs appropriately. Choosing the appropriate type of medication with appropriate dosage strength, with simplified medication regimen and involving caregivers, can lead to a better compliance [7].

In Hungary, data from the single state health insurance-supported financing of prescription medicines are collected in a uniform database. Based on this database, we aimed to analyze the use of ChEis for the treatment of dementia to provide real-life information from the Eastern European region with scarce data on this field.

## Methods

The NEUROHUN database was used in our study. It includes all in- and outpatient medical records (except family medicine) and medication prescription reports from Hungary between 2004 and 2017 [8], using data of redeemed prescriptions (prescription fills). Data from 2013 to 2016 were analyzed in our study. Original patient identifier codes were anonymized and encrypted identifiers were used. All personal data protection regulations were followed. The study was approved by the Ethics Committee of Semmelweis University, Budapest, Hungary (Approval No: SE TUKEB 88/2015).

### Selection of patients and medications

**Fig. 1:**
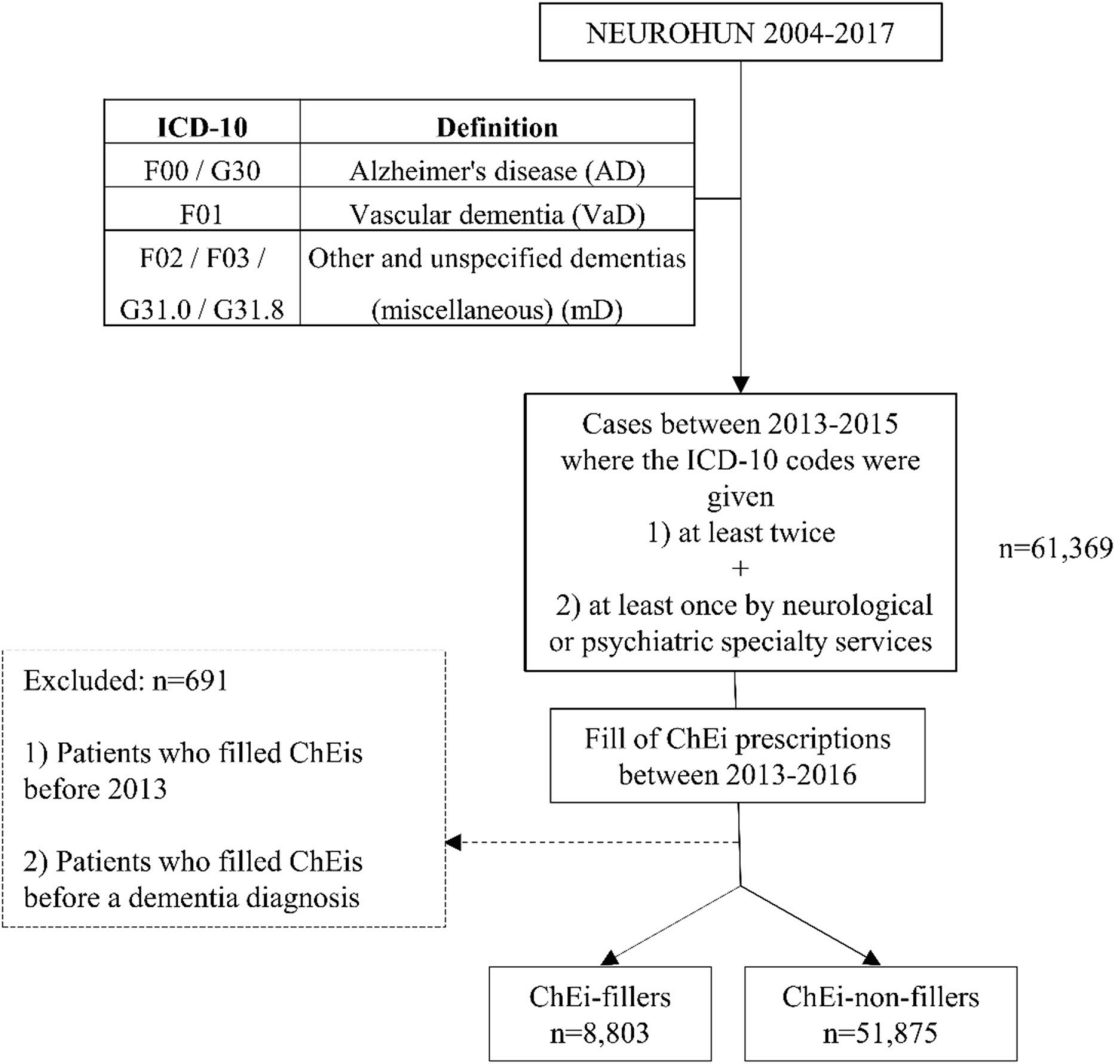
Flowchart of the patient selection

The selection of patients and definitions of dementias were the same as in our previous study [9]. The process is summarized on Fig. 1. Patients with International Classification of Diseases (ICD-10) codes (F00, G30, F01-03, G31.0, G31.8) given at least twice and at least once by neurological or psychiatric specialty services between 2013 and 2015 were selected. Patients receiving dementia diagnoses before 2013 were excluded. The validation of the database was performed as described previously [9]: records of patients were compared in NEUROHUN and the local integrated hospital healthcare information technology system (MedSol, T-System, Hungary) of Semmelweis University, Budapest, in a selected period (October 2013) and medical records were reviewed to ensure that the clinical findings support the diagnosis of dementia.

In Hungary, the marketed ChEis in this period were donepezil (Anatomical Therapeutic Chemical Code (ATC): N06DA02) and rivastigmine (ATC: N06DA03). Donepezil formulations were 5 and 10 mg tablets, with the minimally effective dose of 5 mg once a day. Rivastigmine was available in 3, 4.5, and 6 mg capsules, with the minimally effective dose of 3 mg twice a day. In addition, rivastigmine was also available as a 4.6 and 9.5 mg/24 h patch (the higher dose being the minimally effective daily dose).

In NEUROHUN, all redeemed prescription data are available. We filtered the selected patients for these two ChEis and followed the medication fills between 2013 and 2016, with 1 year as a minimal follow-up (Fig. 1). Patients taking other anti-dementia medications such as nootropic agents (piracetam ATC: N06BX03, vinpocetine ATC: N06BX18, nicergoline ATC: C04AE02 being available in Hungary during the studied period) or memantine (ATC: N06DX01) were also included.

### Analysis of adherence and persistence

**Fig. 2:**
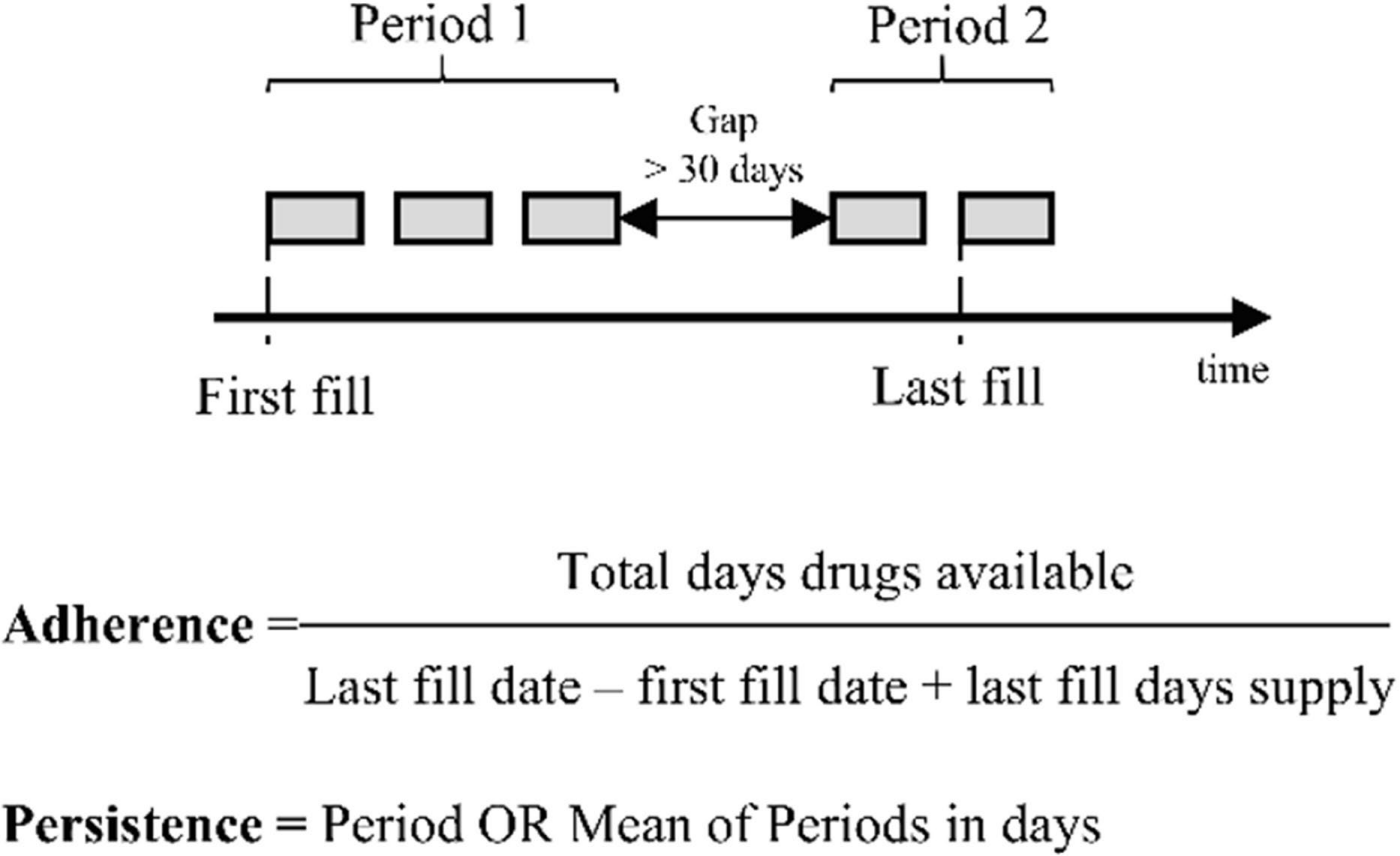
Definition of adherence and persistence

Adherence (Fig. 2) was used to define the degree of redeemed prescriptions in a given interval by the application of the proportion of days covered (PDC) formula. Patients were divided into 3 groups: adherents were those with a PDC of at least 80%, partially adherents with a PDC between 20 and 79%, and patients with a PDC less than 20% were categorized as non-adherent [10].

Persistence (Fig. 2) was applied to represent the duration of time over which a patient continued to fill ChEi prescriptions. In our analysis, the permissible gap (i.e., the threshold to the period of time with absence of treatment) was determined in 30 days [11]. If a patient had gap(s) in its therapy longer than 30 days, the persistence was calculated as the mean of the periods. The median time of persistence in different groups were analyzed with Kaplan–Meier curves and log rank test. The endpoint events were death or discontinuation of ChEi (if it happened at least 30 days before the end of study (12/31/2016)), whichever occurred first.

### Statistical analysis

Descriptive and survival analyses were performed for filler and non-filler populations as well. Data were analyzed according to medications and specialty services (neurological, psychiatric or both) giving the diagnoses.

Microsoft Excel 2016 (Microsoft Corporation, Redmond, Washington, USA) (descriptive statistics), TIBCO Statistica version 13.5 (TIBCO Software Inc, Palo Alto, California, USA) (one-way ANOVAs, multiple logistic regression), and GraphPad Prism 8.0.1 (GraphPad Software Inc., San Diego, California, USA) (Kaplan–Meier survival curves, log rank tests) were used for analyses.

## Results

### Descriptive analysis of data

61,369 patients were included. 8,803 of them filled ChEi prescriptions at least once (ChEi-fillers) during the study period, while 51,875 patients did not fill any (ChEi-non-fillers). Approximately one-third (31.5%) of ChEi-non-fillers did not take any other medication for dementia, 64.2% took at least one of the nootropics, 0.79% took memantine only, and 3.42% took memantine and at least one nootropic. 81.4% of the ChEi-fillers also took at least one nootropic and 24.1% of them filled at least one memantine prescription also.

ChEi-non-fillers had more inpatient records in neurological and psychiatric specialties (mean 1.36 times/patient) and other specialties (4.46 times/patient) compared to ChEi-fillers (1.09 times/patient and 3.44 times/patient, respectively). 7.67% (*n* = 627) of ChEi-fillers were diagnosed without neuroimaging, while the ratio was 36.08% (*n* = 13.753) among ChEi-non-fillers.

ChEi-fillers were divided into donepezil, rivastigmine, and switcher (taking both ChEis in the study period) groups. The characteristics of these groups and the ChEi-non-fillers are summarized in Table 1.

**Table 1:** Summary of basic characteristics of the groups

Group	Donepezil	Rivastigmine	Switcher	ChEi-non-fillers
**Number of patients**	7909	524	370	51,875
Men (%)	2697 (34.1)	217 (41.4)	142 (38.4)	18,465 (35.6)
Women (%)	5212 (65.9)	307 (58.6)	228 (61.6)	33,410 (64.4)
**Mean age (± SD)**^a^	75.98 ± 7.94	76.24 ± 7.81	75.03 ± 7.83	76.97 ± 10.82
**Observed deaths (%)**	1921 (24.3)	158 (30.2)	81 (21.9)	25,651 (49.4)
**Diagnosis given by**				
Neurologist (%)	2650 (33.5)	147 (28.1)	125 (33.8)	9567 (18.4)
Psychiatrist (%)	1867 (23.6)	72 (13.7)	44 (11.9)	27,302 (52.6)
Both (%)	3392 (42.9)	305 (58.2)	201 (54.3)	15,006 (29.0)

^a^The difference between donepezil and switchers vs non-fillers was significant (one-way ANOVA, post-hoc Tukey HSD *p* < 0.002); *SD* standard deviation

Patients diagnosed by psychiatric specialty services only received ChEi in a lower proportion than patients with neurological only or both (neurological and psychiatric) diagnoses. In all ChEi-filler groups, those with diagnoses from both specialty services had higher numbers (42.9–58.2%), but had significantly longer time between the first diagnosis and the first fill (the mean was 134.5 days) compared to patients with only neurological or psychiatric diagnoses (92.3 and 103.6 days, respectively) (one-way ANOVA, post hoc Tukey HSD, *p* < 0.00002). The time between the first diagnosis and the first fill in patients with only neurological and only psychiatric diagnoses was not significantly different.

The proportion of women was higher in all groups. The mean age was the highest in the ChEi-non-filler group, the difference being significant compared to the donepezil and switcher groups only (one-way ANOVA, post-hoc Tukey HSD, *p* < 0.002).

The proportion of the patients with AD diagnosis was 93.7% in donepezil, 95.8% in rivastigmine, and 98.9% in switchers group. ChEi-non-fillers had more VaD or mD diagnoses, with the VaD:AD ratio of 5.1:1 (this ratio was 1:2.3 in ChEi-fillers and 2.5:1 in total).

Donepezil use was far more frequent than rivastigmine, and rivastigmine patch (*n* = 419) was preferred against capsules (*n* = 56); only a few patients used oral and patch forms as well (*n* = 49). Nevertheless, there were 177 patients who used the 4.6 mg rivastigmine patch, without increasing the dose to the recommended therapeutic dose of 9.5 mg patch. Among donepezil users, 16.4% (*n* = 1294) took 5 mg tablets only, 51.8%, (*n* = 4,098) 10 mg only, while both doses were taken by 31.8% (*n* = 2517).

Donepezil was the first choice in 75.7% of switcher (*n* = 280). The mean time until switch in any direction was 188 days, 194 days from donepezil to rivastigmine, and 170 days from rivastigmine to donepezil.

Patients with donepezil monotherapy filled their first prescription after 116.2 days from their first dementia diagnosis, which was not significantly different compared to rivastigmine monotherapy (99.5 days) (one-way ANOVA, post-hoc Tukey HSD, *p* < 0.18).

### Survival analysis

**Fig. 3:**
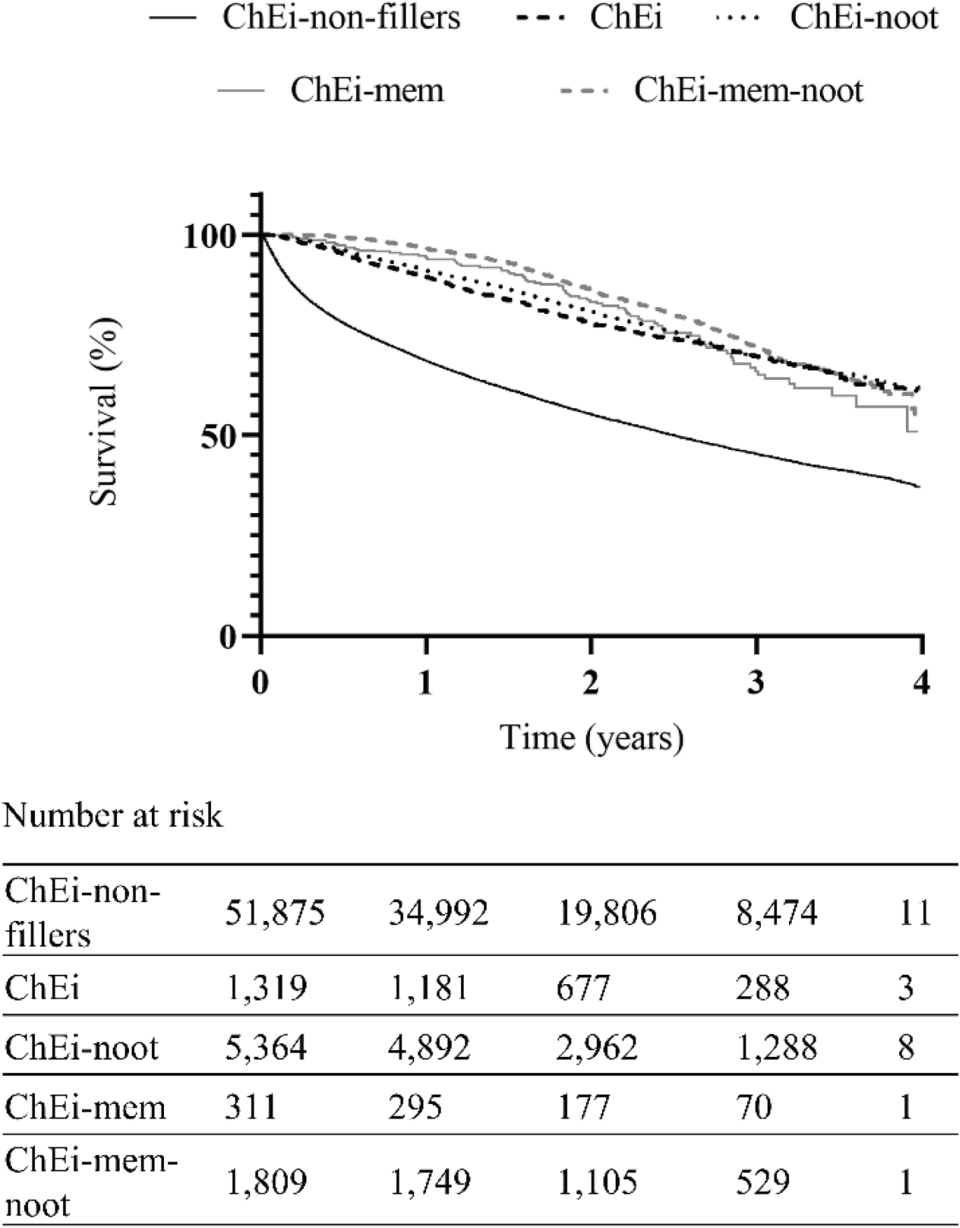
Survival of patients with or without taking ChEis. The median survival of ChEi-non-fillers was 2.50 years. In all groups of the ChEi-fillers, the median survival was more than 4 years (*p* < 0.0001). Among the ChEi-fillers, significant difference was found between ChEi vs ChEi-mem-noot (combination of ChEi, memantine, and nootropics) (*p* < 0.0004) and ChEi-noot vs. ChEi-mem-noot (*p* < 0.0027). (Combined used means that patients were filled with the drugs at least once during the study period, but not necessarily used the drugs simultaneously.) Kaplan–Meier curves, log rank test. In addition to the deceased patients, the number at risk is also decreased by patients whose observation was shorter than the study period. *mem* memantine, *noot* nootropics

The median survival in the ChEi-non-filler group was 2.50 years, while in case of ChEi-fillers, the median could not be determined during the analyzed period, as it was more than 4 years. The median survival was more than 4 years in all groups of ChEi-fillers and the mean survivals based on Kaplan–Meier analyses were significantly different (log rank test, *p* < 0.05), being 3.25 (95% confidence interval (CI95) 3.22–3.27), 3.1 (CI95: 3.00–3.22), and 3.4 (CI95: 3.29–3.51) years for donepezil, rivastigmine, and switcher, respectively. Case fatality and survival were the worst in the ChEi-non-fillers, while the best in switchers (Table 1, Fig. 3).

ChEi use had the strongest positive effect on survival (odds ratio (OR) 2.98, CI95: 2.82–3.14). Female gender was also protective (OR 1.80, CI95: 1.73–1.87), while older patients had the worst case fatality (OR 0.920, CI95: 0.917–0.921) (multiple logistic regression, *p* < 0.00001).

### Adherence and persistence

**Fig. 4:**
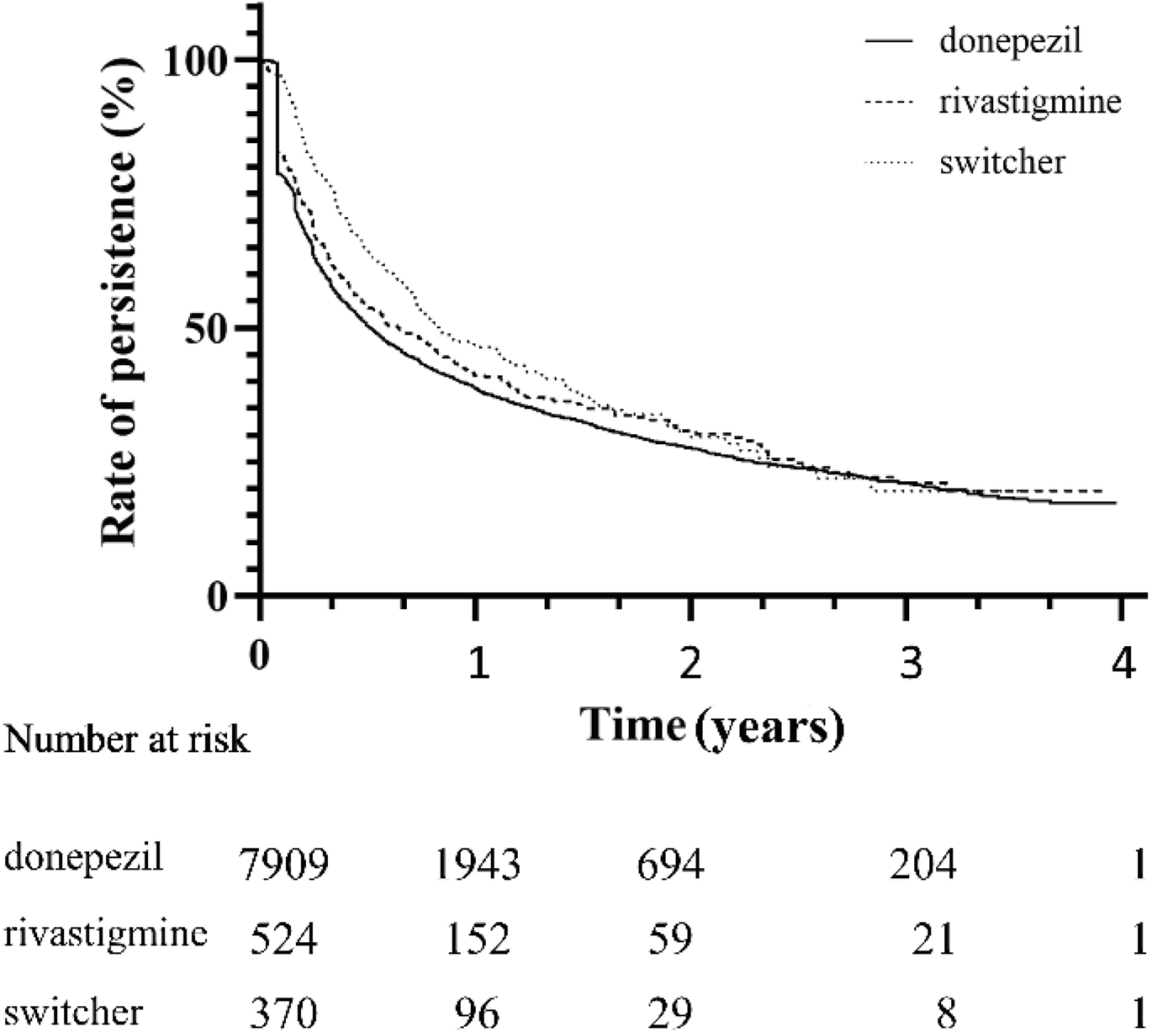
Persistence for ChEis. Kaplan–Meier curves, log rank test, *p* < 0.0001

19.7% of donepezil (*n* = 1561) and 17.0% of rivastigmine (*n* = 89) fillers used ChEis for a maximum of 30 days. Among switchers, there were 14 patients (3.8%) taking ChEis for a maximum of 60 days. The median persistence for ChEis was significantly different in the 3 groups: 188.0 days (CI95: 175.6–200.4) in donepezil, 237.0 days (CI95: 179.0–295.0) in rivastigmine, and 308.0 days (CI95: 227.4–388.6) in switcher. After 1 year, 24.6% of donepezil users, 29.0% of rivastigmine users, and 25.9% of switcher were persistent (Fig. 4).

**Fig. 5:**
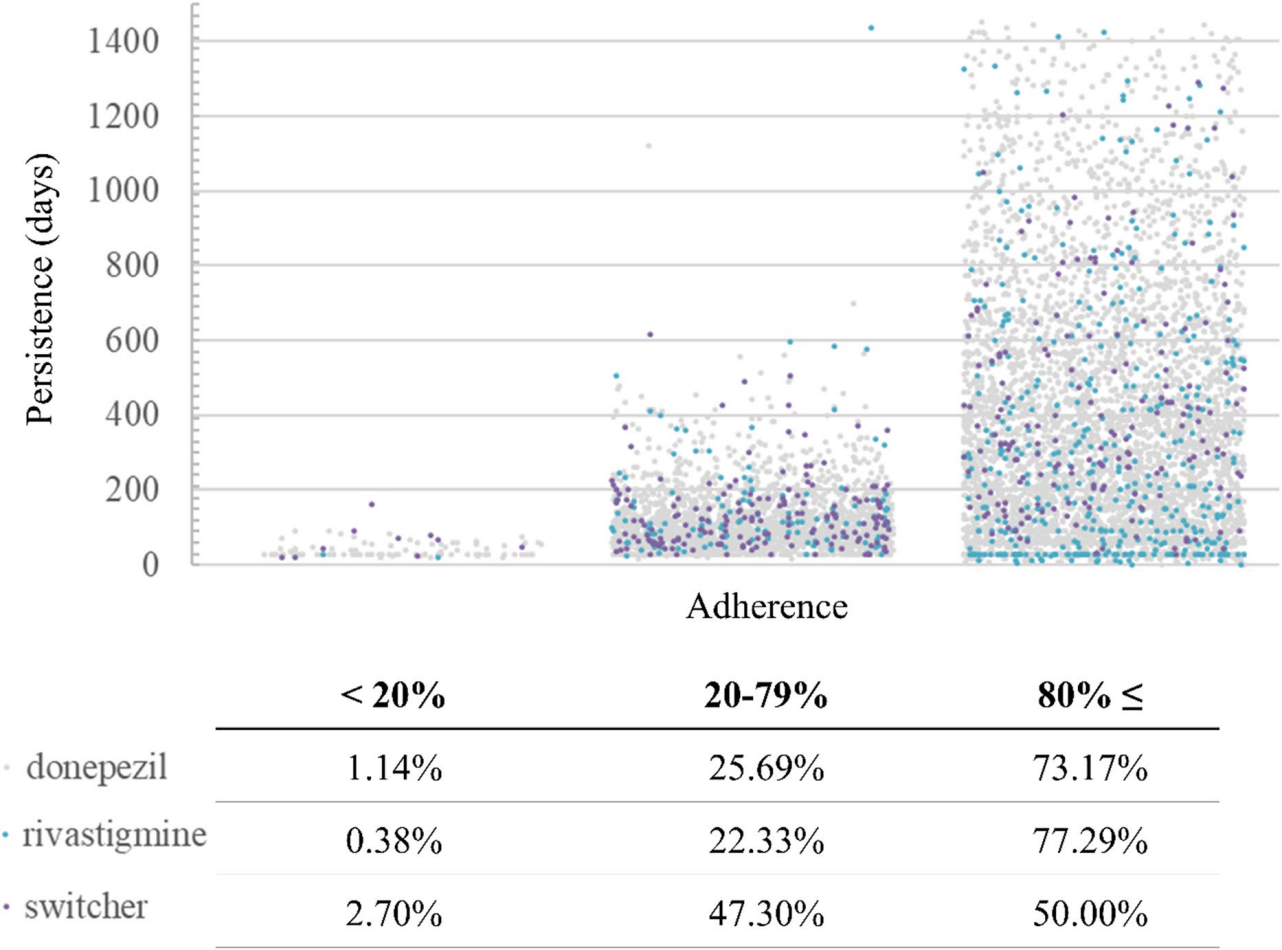
Adherence and persistence of all ChEi filler patients. The adherence was categorized in 3 groups on the *X* axis, while persistence was visualized as a continuous time line in days on the *Y* axis. Each dot represents a patient, donepezil was marked with grey, rivastigmine with blue, and switcher with purple

Non-adherent patients also had a short persistence, while adherent patients had longer prescription-filling durations. The adherence to donepezil and rivastigmine was similar, while in the switcher group, the proportion of partially adherent patients was higher (Fig. 5).

In accordance with the ratios in the adherence, the amount of gaps was significantly higher (*p* < 0.00002) in switcher (mean number of the gaps 1.39, while 0.71 and 0.57 in donepezil and rivastigmine groups, respectively). In addition, the duration of the gaps was significantly different, being 136.3 days for switchers, 98.2 days for donepezil, and 70.3 days for rivastigmine users (one-way ANOVA, post-hoc Tukey HSD *p* < 0.01).

Donepezil and rivastigmine data were analyzed separately as well in the switcher group and no significant difference was found between the median persistence of donepezil (92 days) and rivastigmine (120 days) (log rank test *p* = 0.059). Adherence ratios were similar to monotherapy groups: for donepezil, the ratio of partially adherents was 24.6% and the adherents was 74.9%, while for rivastigmine these were 20.8% and 78.9%, respectively.

In the rivastigmine monotherapy group, patch and capsule groups were also compared. There was no significant difference between the median persistence for rivastigmine patch (217 days) and capsule (194 days) (log rank test *p* = 0.09), while the adherence was better for the patch with the ratio of partially adherents of 14.3% and adherents of 85.4% versus 53.6% and 44.6% for capsule, respectively.

In the donepezil group, the worst median persistence (60 days) and the best adherence (partially adherent 14.3%, adherent 87.2%) was observed in patients filling only the 5 mg tablets. Median persistence of patients filling only the 10 mg tablets was 150 days, while patients filling both the 5 and 10 mg tablets had the best median persistence (428 days). In the last two groups, the adherence was the same (28.8% and 70.1% vs. 28.0% and 70.9% for partially adherent and adherent patients, respectively) (log rank test *p* < 0.0001). 48.0% of the 5 mg only group filled only one prescription during the 4-year study period, while this proportion was 26.5% in the 10 mg only group and 2.54% in the 5 and 10 mg filling group.

## Discussion

During the 4-year period, only 14.5% of patients with a dementia diagnosis (not necessarily AD) filled at least one ChEi prescription. 94.0% of ChEi-fillers had AD diagnosis. Although patients with different subtypes of dementia diagnoses were selected in our study, the prescriptions showed adherence to the approved indication of AD and were hardly used in off-label indications such as VaD or Lewy-body dementia. In Hungary, ChEi prescriptions are reimbursed only with AD diagnosis (G30.90), which is probably the cause of the different VaD:AD ratios between the ChEi-fillers (1:2.3) and ChEi-non-fillers (5.1:1) groups.

In the USA, approximately 50% of patients diagnosed with dementia received antidementia medications, but those who were diagnosed in a later stage of dementia were treated less frequently [12]. In Spain, antidementia pharmacotherapy occurred in 70.4% of patients diagnosed with AD [13]. Medication use among dementia patients as low as in our current study was seen in the UK in 2005, but the proportion there doubled during the next 10 years [14]. In Austria, the annual prevalence of antidementia medications use was 1% [15], while this was 0.09% in Hungary over the whole 4-year period in our study, in spite of the fact that the prevalence of dementia was just 1.33 times higher in Austria [16].

In our study, the mean age of ChEi-non-fillers was the highest (although the difference was only approximately 1 year), similar to the USA [12], which might partially contribute to the low rate of prescriptions in this group. In addition, the strict national regulation of ChEi-prescription could also influence the availability of antidementia drugs: the diagnosis of dementia and prescriptions of ChEis must be made by a neurologist or a psychiatrist and continued prescription depends on the progression rate of the disease measured on the Mini Mental State Examination (MMSE) test.

The proportion of patients receiving ChEis was the highest in the group where the diagnosis was given by neurological and psychiatric services too, but with a significant delay in the initiation of the therapy. The least proportion of patients taking ChEis was seen in the group with the psychiatric diagnosis only.

Two-thirds of the ChEi-non-fillers took nootropics. Piracetam, vinpocetine, and nicergoline have documented positive effects on cognitive disorders (see reviews of Winblad [17], Zhang [18], and Saletu et al. [19]); moreover, according to a Taiwanese study [20], piracetam, gingko, and some ergot derivates can prolong the survival of patients with degenerative and vascular dementia. Despite these effects, nootropics might be only adjuvant therapies next to the first-line ChEis in AD [21].

In our study, the survival of ChEi-filler patients was significantly longer than in ChEi-non-fillers. The survival was also significantly longer in patients taking only nootropic drugs than in patients with no medication (data not shown). Multivariate analysis confirmed that ChEi-filling was an independent significant predictor, even after data were adjusted to age and gender as well. In Hungary, the median survival of patients with all dementias was 3.01 years [9]; in comparison, ChEi-non-fillers had 2.50 years, while in case of ChEi-fillers it was more than 4 years. There was no significant difference in the length of survival between only ChEi and ChEi plus memantine users, but detailed analysis of memantine use was not the aim of our present study. The better survival observed with ChEi-use was also confirmed by Swedish results of Wattmo et al. [22], where better survival was expected with higher doses and longer continuous medication periods.

It could be hypothesized that ChEi-fillers had a more consistent medical attention, which is also supported by the more common use of neuroimaging among them. In addition to the hardly but significantly lower mean age of ChEi-fillers, these patients might be in earlier stages of dementia, suggested by the fewer inpatient appearances.

In addition to the longer survival, case-fatality of ChEi-fillers was nearly half of the ChEi-non-filler group (24.5% vs. 49.4%, respectively) in our study, ChEi use causing the strongest positive effect (OR 2.98, CI95: 2.82–3.14); although other confounding factors such as co-morbidities were not considered. In a study of Mueller et al. [23], however, a 40% reduction in mortality was observed and it remained significant after adjusting for all covariates such as age, gender, demographic factors, and cognitive function at baseline. In our study, there were fifteen times more donepezil users than rivastigmine. In Poland, donepezil use was three times more common [24], while in Taiwan and Austria the ratio was even more balanced; donepezil was used only 1.4 and 1.16 times more than rivastigmine, respectively [15, 25]. The considerable difference in Hungary may be partially due to the fact that donepezil is available in several generic forms in contrast to rivastigmine, which is marketed as an originator only, resulting in considerably higher monthly therapeutic expenses.

Nearly 20% of patients did not fill further prescription after one month both in the donepezil and rivastigmine groups, the ratio being similar as seen in Austria (14%) [15]. Differently from previous studies [11, 24, 25], where the follow-up of patients was stopped after their drug discontinuations (gaps), we followed discontinuing patients and found that the majority of them returned to ChEi-therapy with a partial or good adherence, which finding is of practical importance in clinical practice.

The best compliance was associated with rivastigmine patch, similarly to the findings of Molinuevo et al. [26], while in Taiwan [25] oral form of rivastigmine and donepezil were better.

In a Polish study [24], the 1-year persistence for donepezil was 42.2%; for rivastigmine, it was 46.0%, allowing a 90-day gap. Considering our results, the 1-year persistence was 24.6% for donepezil and 29.0% for rivastigmine, however allowing only 30 days gaps, but with possible restart of the therapy. Although comparison is difficult, the persistence of rivastigmine seems better. Our results are similar to those in Korea [11], the 1-year persistence for ChEi with a 30-day gap being 24.0%.

Donepezil users redeeming both 5 and 10 mg tablets had the highest persistence, with its median of 1.17 year. Presumably because of the dose titration needing both the 5 and 10 mg tablets, patients and their caregivers might had been better informed and possible side effects leading to treatment discontinuation might had been less frequent.

The median persistence for ChEis was longer in patients treated with both agents compared to the monotherapy groups, although their adherence was worse, with higher proportion of partially adherents. Their mean age and time from first diagnosis to first fill did not differ significantly from both monotherapy group, but their mean survival and case fatality were the best in this population. Based on these, it arises that these patients may have filled their first prescriptions with a milder onset of symptoms and the switch occurred when symptoms worsened.

The high rate of partially adherent patients among monotherapy (22–26%) and switcher (47%) groups suggests that a more thorough education of these patients and their caregivers might be effective to improve their compliance.

42.9% of ChEi-fillers (not including deceased patients) discontinued the therapy and only approximately a quarter of ChEi-fillers (26.5%) tried to change medication to at least once to the other available ChEi or memantine. In addition, the high proportion of nootropic use might indicate that prescribers are not tightly adherent to treatment guidelines prioritizing the use of ChEis and memantine for the treatment of AD [27].

### Limitations and strengths of the study

The NEUROHUN database allows us to analyze prescription refill records of ChEis in dementia diagnosed population. There is a linkage of clinical records with medication refill data on a patient-by-patient basis. The strict criteria for dementia diagnosis helped to improve specificity, but at the price of a lower sensitivity. In our study, the education levels, the socioeconomical status and co-morbidities (such as hypertension or other cardiovascular risk factors) were not considered but their effects were indirectly examined. Due to the nature of the study, examination of individual patients could not be performed, and their detailed cognitive status was unknown. However, the nationwide database covering the whole Hungarian population provides opportunity to a long follow-up of a large sample.

## Data Availability

Data transparency: data used for the analysis in our study will be provided in an Excel file as a supplementary/supporting file for Geroscience after acceptance or for the review process.

## Code availability

Software application or custom code: not applicable.

## Declarations

Additional declarations for articles in life science journals that report the results of studies involving humans and/or animals are not applicable.
